# Investigating the Combined Effects of Mechanical Stress and Nutrition on Muscle Hypertrophic Signals Using Contractile 3D-Engineered Muscle (3D-EM)

**DOI:** 10.3390/nu15184083

**Published:** 2023-09-21

**Authors:** Dong Yi, Takeshi Sugimoto, Teppei Matsumura, Sho Yokoyama, Toshia Fujisato, Tomohiro Nakamura, Takeshi Hashimoto

**Affiliations:** 1 Faculty of Sport and Health Sciences, Ritsumeikan University, 1-1-1 Nojihigashi, Kusatsu 525-8577, Shiga, Japan; anotherd2018@gmail.com (D.Y.); hadano4249@gmail.com (T.S.); sh0207hs@ed.ritsumei.ac.jp (T.M.); 2 Department of Mechanical Engineering, School of Engineering, Osaka Institute of Technology, 5-16-1 Omiya, Osaka 535-8585, Osaka, Japan; sho.yokoyama@oit.ac.jp; 3 Graduate Course in Applied Chemistry, Environmental and Biomedical Engineering, Osaka Institute of Technology, 5-16-1 Omiya, Osaka 535-8585, Osaka, Japan; toshiya.fujisato@oit.ac.jp; 4 Division of Human Sciences, Faculty of Engineering, Osaka Institute of Technology, 5-16-1 Omiya, Osaka 535-8585, Osaka, Japan; tomohiro.nakamura@oit.ac.jp

**Keywords:** 3D-EM, maca, EPS, hypertrophy, sarcopenia

## Abstract

Since 3D-EM closely resembles in vivo muscles, the aim of this study was to investigate the effects of exercise (electrical pulse stimulation (EPS)) and nutrition (maca), which contains triterpenes, on muscle hypertrophy by using 3D-EM for the first time. The 3D-EM was composed of C2C12 cells and type 1 collagen gel, was differentiated for 14 days, and was divided into four groups: control, maca, EPS, and maca + EPS. The medium was replaced every two days before each EPS intervention, and the concentration of maca in the culture solution was 1 mg/mL. The intervention conditions of the EPS were 30 V, 1 Hz, and 2 ms (24 h on, 24 h off, for one week). The expression levels of proteins were examined by Western blotting. The intervention of maca and EPS upregulated the expression of MHC-fast/slow (both *p* < 0.05) compared with the control group, and the addition of maca had no effect on the phosphorylation of mTOR (*p* = 0.287) but increased the AMPK phosphorylation (*p* = 0.001). These findings suggest that intervention with maca and EPS has a positive effect on muscle hypertrophy, which has a positive impact on sarcopenia. However, the underlying mechanisms remain to be further explored.

## 1. Introduction

Sarcopenia is a geriatric muscle disease characterized by low muscle mass, low muscle strength, and low physical performance, and it also leads to disabilities and an increased risk of death [1]. In addition to aging, disease, a sedentary lifestyle, energy deficiency, and poor protein intake can also lead to an increased incidence of sarcopenia [2,3]. However, resistance exercise and nutrition are known to counteract muscle wasting [4,5].

Regarding nutrition, numerous plant-derived natural compounds have demonstrated their capacity to induce muscle growth and hypertrophy, including curcumin, resveratrol, green tea, and ginkgo biloba extract [6,7,8]. Among them, over the past 20 years, there has been a growing interest in and demand for Lepidium meyenii Walp, or maca, due to its health benefits [9]. Recent studies indicate that maca may improve endurance, help with autism, and have anti-inflammatory and anticancer properties [10,11,12,13]. Maca’s secondary metabolites provide neuroprotective and cytoprotective benefits [14] and improve energy metabolism [15]. Notably, triterpenoid saponins found in maca, such as panaxatriol, have been suggested to have a muscle hypertrophy effect [16] that increases muscle protein synthesis (MPS) [17]. In addition to triterpenoid saponins, maca contains essential nutrients and various amino acids, such as leucine and arginine [18], which are associated with promoting muscle growth. [19,20,21]. Indeed, our recent research showed that maca can induce muscle hypertrophy in C2C12 skeletal muscle cells [22].

Exercise can treat and prevent various diseases, possibly by contractile skeletal muscles producing and releasing signaling molecules [23]. Usually, electrical pulse stimulation (EPS) is used to mimic exercise (i.e., mechanical stress on skeletal muscle) and investigate underlying mechanisms in both experimental animal and cell culture models [24]. EPS is utilized as a treatment against muscle atrophy as a result of muscle disuse in in vivo models [25]. It has been reported that in human cultured muscle cells, EPS effectively promotes myotube hypertrophy and increases MPS activity [26,27].

Usually, a combination of nutrition (e.g., amino acid) and resistance exercise shows synergic effects on muscle growth and hypertrophy [28]. To further substantiate the impact of combining nutritional and exercise (i.e., mechanical stress on skeletal muscle) interventions on muscle hypertrophic signaling through cellular experiments, animal experiments are utilized for validation [29,30]. Nevertheless, complexities arise when targeting specific tissues, such as muscle tissue, due to individual variations and the influence of hormones on organisms, making cell culture-based experiments particularly important from the perspective of ethically reducing animal use and increasing the efficiency of clinical development [31]. To the best of our knowledge, however, there is a lack of research on combining exercise and nutrition in two-dimensional (2D) skeletal muscle cells. Furthermore, although a certain number of studies investigated the sole effect of nutrition or exercise, respectively, on 2D skeletal muscle cells as aforementioned, there is still a gap between in vivo skeletal muscle tissue and 2D skeletal muscle cells in terms of structure and function [32,33].

In recent research, engineered muscles have been used to measure muscle contraction force [34]. Three-dimensional-engineered muscle (3D-EM) structure consists of tightly packed, parallel-oriented bundles resembling myofibers, thereby forming sarcomere structures akin to those observed in living muscle tissue. In addition, 3D-EM has been reported to have a 10-fold higher maximal twitch tension compared to that of C2C12 myotubes in 2D culture [35]. Thus, the 3D-EM might provide a close representation of muscles in living organisms. In this regard, we recently identified contractile muscle-derived myokine secreted by EPS-evoked muscle contractile stimulation in 3D-EM [36].

Therefore, the aim of this study was to examine the combined effect of nutrition (i.e., maca) and mechanical stress (i.e., EPS) on muscle growth and hypertrophy in 3D-EM, a better model for mimicking living muscle, for the first time.

## 2. Materials and Methods

### 2.1. C2C12 Cell Culture

C2C12 skeletal muscle cells (American Type Culture Collection, Manassas, VA, USA) were cultured in growth medium (GM), which was composed of Dulbecco’s modified Eagle’s medium (DMEM with high glucose (4.5 g glucose/L); Nacalai Tesque, Kyoto, Japan) supplemented with 10% fetal bovine serum (FBS; Sigma—Aldrich, St. Louis, MO, USA), along with 100 U/mL penicillin and 100 μg/mL streptomycin (PS; Nacalai Tesque, Kyoto, Japan). The culture medium was replaced every 48 h, and all experimental cultures were conducted within a humidified incubator set at 37 °C with 5% CO_2_ [22,37,38,39].

### 2.2. The Manufacture of 3D-EM

**Figure 1:**
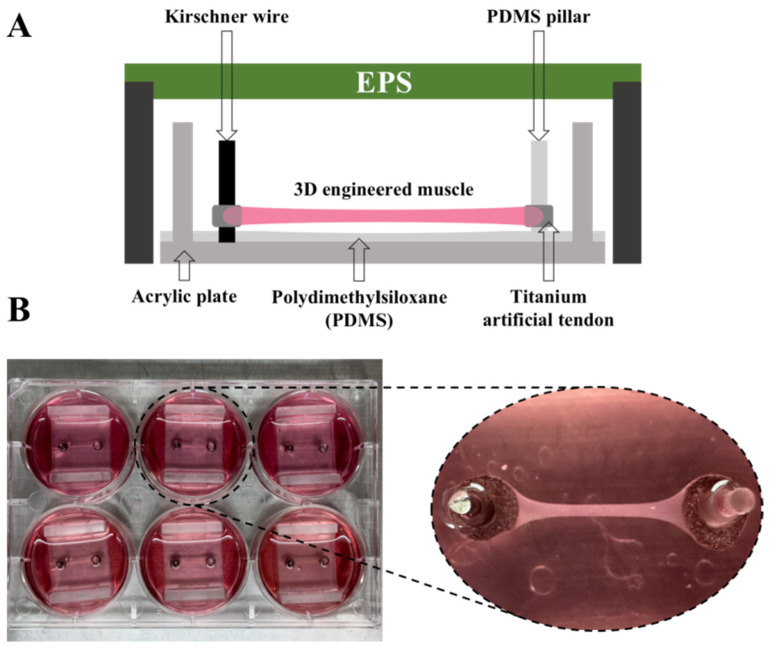
(**A**) Titanium artificial tendons were inserted into Kirschner wire and PDMS pillars. C2C12 myoblasts were embedded in a cold type-I collagen gel solution at a density of 1.5 × 10^7^ cells/mL. A 100 μL suspension of C2C12 cells was added between two artificial tendons. (**B**) Top view of 3D-EM after 14 days of differentiation.

The 3D-EM was produced following previously reported methods [35,40]. Through Harada’s modification of this device, a unique pillar was made of PDMS material on one side [41]. An artificial tendon made of titanium scaffold (Zellez™; Hi-Lex Corporation, Tokyo, Japan) was employed (Figure 1A). C2C12 cells were embedded in a cold type-I collagen gel solution (Cellmatrix; Nitta Gelatin, Osaka, Japan) at a density of 1.5 × 10^7^ cells/mL. A 100 μL cold suspension of C2C12 cells was added between two artificial tendons, and after incubation for 30 min at 37 °C for gelation, the constructs were placed in GM and incubated for 2 days at 37 °C and 5% CO_2_. In lieu of GM, we employed a differentiation medium (DM), which was composed of high-glucose (4.5 g glucose/L) DMEM containing 7% horse serum (HS; Thermo Fisher Scientific, Tokyo, Japan) and 1% PS (Nacalai Tesque, Kyoto, Japan). The cells were cultured for a duration of 14 days for myoblast differentiation into myotubes in 6-well plates (Figure 1B). The structure and function can be supported by the 3D-EM morphology observed in a previous study [35], where contraction of the 3D-EM was observed when cells were stimulated [36].

### 2.3. Experimental Design

On Day 14 of differentiation, the 3D-EM was divided into 4 groups. The control (con) group (*n* = 3), maca group (*n* = 3), EPS group (*n* = 3), and maca + EPS group (*n* = 3). Three independent experiments were performed such that the total number of samples was *n* = 9 in each group. The con group was incubated at standard conditions. The maca group and maca + EPS group were treated with 1 mg/mL maca for one week. The maca extract was solubilized in dimethyl sulfoxide (DMSO; Wako, Kyoto, Japan) and subsequently blended with the DM. To keep the growth conditions consistent for the maca-supplemented groups, we added DM containing the same amount of DMSO as the maca-treated groups to the con and EPS groups. The medium was changed every two days.

### 2.4. Electrical Pulse Stimulation (EPS)

Following each medium change, we applied EPS to the 3D-EM within a C-Dish (Ion Optix, Westwood, MA, USA), and the EPS was administered using the C-Pace electrical stimulation generator (Ion Optix, Westwood, MA, USA) for a duration of 24 h under contractile stimulation conditions (30 V, 1 Hz, 2 ms). Subsequently, we allowed the 3D-EM to rest for 24 h and repeat this work for a week. Since EPS-induced hypertrophy occurred during the resting period after cessation of EPS [26], we made some modifications to the methods used in the previous study exploring hypertrophy-causing EPS conditions in 2D skeletal muscle cells [42], with the extension of 3D-EM differentiation time [35].

### 2.5. Western Blot Analysis

After the last 24 h of EPS intervention on the 3D-EM, it was washed twice with PBS, separated from the tendon, pulverized with a power masher (Nippi, Tokyo, Japan) for 1 min in RIPA buffer with protease and phosphatase inhibitors, and then pulverized with an ultrasonic dispersion machine (UH-50; SMT, Tokyo, Japan) for 1 min. After incubating the samples on ice for one hour, we subjected them to centrifugation at 15,000× *g* for 15 min at 4 °C. To verify the concentration, we collected the supernatant and performed an analysis using a Wako protein assay kit (FUJIFILM Wako Pure Chemical Corporation, Osaka, Japan).

Equal quantities of proteins were segregated on 8% SDS-polyacrylamide gels, applying a constant current of 40 mA for 1 h. Subsequently, they were transferred onto polyvinylidene fluoride membranes at 60 V for 2 h. Blocking One-P and Blocking One solution (Nacalai Tesque, Kyoto, Japan) were applied for blocking at room temperature following the previously established protocol [22,43], for a duration of 30 min.

Following the blocking step, the membranes were subjected to incubation with primary antibodies against various targets, each at its respective dilution: proteins associated with muscle growth (MHC-fast (Sigma-Aldrich, St. Louis, MO, USA; 1:1000; M4276) and MHC-slow (Abcam, Cambridge, MA, USA; 1:1000; ab11083)), proteins associated with MPS and muscle protein breakdown (MPB) (phosphorylated-mTOR (Ser2448) (Cell Signaling Technology, Danvers, MA, USA; 1:1000; #5536), mTOR (Cell Signaling Technology, Danvers, MA, USA; 1:1000; #2983), phosphorylated-Akt (Thr308) (Cell Signaling Technology, Danvers, MA, USA; 1:1000; #9275), Akt (Cell Signaling Technology, Danvers, MA, USA; 1:1000; #9272), phosphorylated-p70S6K (Thr389) (Cell Signaling Technology, Danvers, MA, USA; 1:1000; #9205), p70S6K (Cell Signaling Technology, Danvers, MA, USA; 1:1000; #2708), phosphorylated-p44/42 MAPK (Erk1/2) (Thr202/Tyr204) (Cell Signaling Technology, Danvers, MA, USA; 1:1000; #4376), p44/42 MAPK (Erk1/2) (Cell Signaling Technology, Danvers, MA, USA; 1:2000; #4696), phosphorylated-4E-BP-1 (Thr37/46) (Cell Signaling Technology, Danvers, MA, USA; 1:1000; #9459), 4E-BP-1 (Cell Signaling Technology, Danvers, MA, USA; 1:1000; #9644), JunB (Abcam, Cambridge, MA, USA; 1:200; ab128878), YAP (Cell Signaling Technology, Danvers, MA, USA; 1:1000; #4912), TRIM63 (MuRF-1) (Santa Cruz, Dallas, TX, USA; 1:100; sc-398608), and MAFbx (Santa Cruz, Dallas, TX, USA; 1:100; sc-166806)), proteins associated with muscle metabolic function (phosphorylated-AMPK (Thr172) (Cell Signaling Technology, Danvers, MA, USA; 1:1000; # 2535) and AMPK (Cell Signaling Technology, Danvers, MA, USA; 1:1000; #2793)), and GAPDH (Sigma-Aldrich, St. Louis, MO, USA; 1:10,000; G9545).

After a series of three washes, each lasting for 10 min with Tris-buffered saline-Tween 20 (TBST), the membranes underwent an incubation step with anti-mouse IgG (1:10,000) and anti-rabbit IgG (1:10,000) for the detection of primary antibody binding. Following this, the membranes were subjected to three additional 10 min washes with TBST. To visualize the protein bands, the membranes were treated with the Luminata Forte Western HRP substrate (Millipore Corporation, Billerica, MA, USA), and the bands were visualized using the FUSION FX7 EDGE (Vilber Lourmat, Collégien, France). Then, ImageJ software version 13.0.6 (National Institutes of Health, Bethesda, MD, USA) was utilized for the quantification of band intensities.

### 2.6. Statistical Analysis

The results are expressed as the mean ± standard deviation (SD). Statistical analysis was performed using a one-way analysis of variance (ANOVA). If ANOVA yielded significant results, post hoc comparisons between individual pairs of groups were performed using Tukey’s test. IBM SPSS statistics version 29.0.0.0 (241) was employed for all statistical analyses. A value of *p* < 0.05 was considered statistically significant.

## 3. Results

### 3.1. The Effect of Combining Exercise (i.e., EPS) and Nutrition (i.e., Maca) on the Expression of Proteins Associated with Muscle Growth in 3D-EM

**Figure 2:**
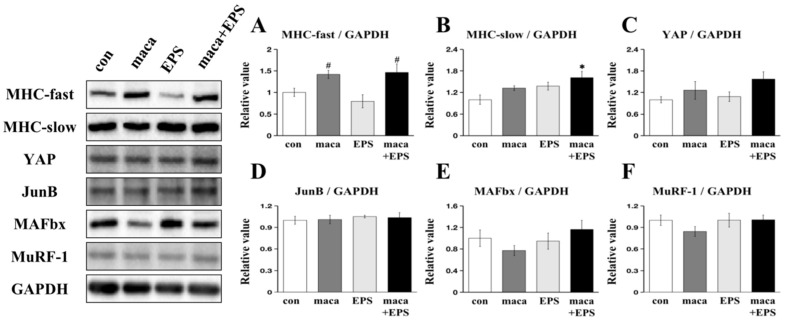
We compared the protein expression levels within the 3D-EM (*n* = 9). The left side displays representative immunoblots, while the quantification of MHC-fast (**A**), MHC-slow (**B**), YAP (**C**), JunB (**D**), MAFbx (**E**), and MuRF-1 (**F**), which was determined after normalization to GAPDH, is presented on the right. At least two independent experiments were performed. *: significant difference from the con group (*p* < 0.05). #: significant difference from the EPS group (*p* < 0.05).

There was a significant main effect of condition on the expression level of MHC-fast (*p* = 0.013), and post hoc comparisons showed that the expression of MHC-fast was significantly higher in both the maca group (*p* = 0.019) and the maca + EPS group (*p* = 0.010) compared to the EPS group, while there was no significant distinction between the con group and EPS group (Figure 2A). In the case of the expression of MHC-slow, there was a significant main effect of condition (*p* = 0.016), and post hoc comparison showed that there was a significant difference between the con group and the maca + EPS group (*p* = 0.009) (Figure 2B). Yet, there were no significant main effects of condition in the expression of the MPS-associated proteins YAP (*p* = 0.127) and JunB (*p* = 0.903) (Figure 2C,D). No significant main effects of condition in the expression of the MPB-related proteins MAFbx (*p* = 0.317) and MuRF-1 (*p* = 0.127) were observed (Figure 2E,F).

### 3.2. Changes in Intracellular Signaling Related to Muscle Protein Synthesis and Metabolism

**Figure 3:**
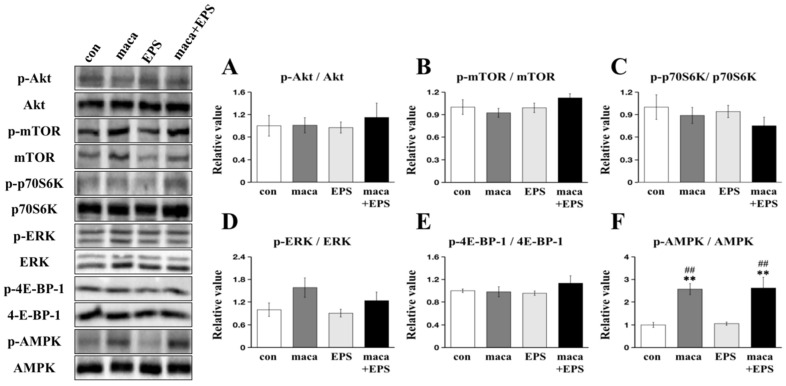
We conducted a comparison of protein expression within the 3D-EM (*n* = 9). The left side displays representative immunoblots, while the quantification of Akt (**A**), mTOR (**B**), p70S6K (**C**), ERK (**D**), 4E-BP-1 (**E**), and AMPK (**F**) phosphorylation compared between the con group, the EPS group, the maca group, and the maca + EPS group is presented on the right. **: significant difference from the con group (*p* < 0.01). ##: significant difference from the EPS group (*p* < 0.01).

No significant main effects of condition were observed in the phosphorylation of Akt (*p* = 0.897) (Figure 3A), mTOR (*p* = 0.287) (Figure 3B), p70S6K (*p* = 0.516) (Figure 3C), ERK (*p* = 0.101) (Figure 3D), or 4E-BP-1 (*p* = 0.415) (Figure 3E). There was a significant main effect of condition on the phosphorylation of AMPK (*p* < 0.001), and post hoc comparisons showed that the maca group and the maca + EPS group were significantly higher than that in the con group and EPS group (all *p* < 0.01) (Figure 3F).

## 4. Discussion

This study is the first to use 3D-EM to investigate the combined effects of nutrition (i.e., maca) and exercise (i.e., EPS) on changes in muscle growth and hypertrophy. In previous research, we found that maca promoted muscle hypertrophy, differentiation, and growth [22], Therefore, we postulated that combining EPS with maca would further promote muscle cell hypertrophy. Our findings indicate that the combination of maca and EPS upregulated the expression of MHC-fast/slow, and the addition of maca increased the phosphorylation of AMPK in 3D-EM.

Exercise and nutrition are known as effective ways to combat muscle wasting and reduce the incidence of many chronic diseases, such as chronic kidney disease, stroke, and chronic heart failure [44,45,46]. However, the effect of the combination of muscle contraction and nutrition on muscle cells is unclear, and from the perspective of reducing the use of experimental animals [47], in vitro 3D models have attracted attention in recent years [48,49]. Collectively, exploring the combined impact of exercise and nutrition in vitro, particularly within 3D models like the 3D-EM, holds significant importance. Therefore, in the present study, we utilized 3D-EM instead of 2D-skeletal muscle cells for the first time to examine the effect of the combination of EPS and maca on muscle hypertrophy.

EPS, as an in vitro exercise model, has previously been used in muscle cells [26]. A low frequency of EPS can upregulate the protein expression of the slow fiber type marker (MHC-slow) in human skeletal muscle cells [42] and promote MPS, leading to muscle hypertrophy [50]. In our study, it was found that low-frequency EPS and maca intervention upregulated the expression of MHC-slow compared with that in the con group (Figure 2B). For the EPS group and the maca group, we did not find any significant changes compared with the con group (Figure 2B). Hence, our proposition is that maca seems to enhance the influence of EPS on MHC-slow expression in the 3D-EM. Within the scope of this investigation, it was also observed that the inclusion of maca led to an increase in MHC-fast expression under EPS conditions, although there was no significant difference in MHC-fast expression between the EPS group and the con group (Figure 2A). Disproportionate atrophy of type IIa muscle fibers (MHC-fast) with aging is well known as a hallmark of sarcopenia [51]. Therefore, we believe that the effect of maca on the expression of MHC-fast (Figure 2A) can provide a new direction for the treatment of sarcopenia. A previous study has shown that the increase in MHC expression levels is accompanied by the occurrence of muscle hypertrophy [52]. We believe that the combination of maca and EPS will also trigger the occurrence of muscle hypertrophy.

Triterpenoids can upregulate the expression of MPS-related proteins and downregulate the expression of MPB-related proteins [53,54,55,56]. In our previous study, we showed that maca could induce hypertrophy in C2C12 skeletal muscle cells [22], and EPS has been previously shown to increase skeletal muscle hypertrophy and metabolic flux in 2D skeletal muscle cells [57]. Therefore, in this experiment, we investigated for the first time the joint effect of exercise (i.e., EPS) and nutrition (i.e., maca) on muscle cell growth in 3D-EM. In line with the findings from our previous research [22], no significant distinction in the expression of proteins related to MPB was observed in the maca group, the EPS group, or the combination of maca + EPS group in this study (Figure 2E,F).

Regarding the signaling pathways related to MPS, the Akt-mTOR pathway and its downstream targets, p70S6K and 4E-BP-1, were not significantly different among the groups (Figure 3A–E). We then further explored the possibility that there might be pathways that promote muscle hypertrophy that are independent of the Akt-mTOR pathway, such as those that involve YAP and JunB [58,59]. Nevertheless, there was also a lack of significant differences in the expression of YAP and JunB. (Figure 2C,D). Our previous study did not observe the upregulation of MPS-related protein expression or the downregulation of MPB-related protein expression, but we observed a positive effect of maca on muscle hypertrophy [22]. Similarly, in this study, we did not observe changes in the expression of proteins related to MPS and MPB. In the 3D tissue-engineered human muscles, compared with 1 Hz of EPS, 10 Hz of EPS induced more expression of MPS-related proteins, thereby causing greater myofiber hypertrophy [27]. Therefore, we think this difference may be due to the use of a lower frequency of EPS (1 Hz) in our protocol, and there is still a gap between human muscles and C2C12 skeletal muscle cells, although the 3D-EM is structurally more similar to living muscle than 2D skeletal muscle cells [35]. Regarding the mechanism, we suggest that further research is needed, and numerous experiments are still needed to verify these results [60].

In this study, no effect of EPS on AMPK phosphorylation was observed, but consistent with our previous study [22], we observed an effect of maca on AMPK phosphorylation (Figure 3F). AMPK promotes mitochondrial biogenesis to provide energy for tissues and cells by regulating the transcriptional activity of PGC-1α, thereby improving chronic fatigue in mouse skeletal muscle [61]. Moreover, AMPK activation is usually accompanied by MPB activation and MPS inhibition via the downregulation of the mTOR pathway [62]. Interestingly, however, phosphorylation of AMPK did not affect MPB- and MPS-related protein expression, as shown in our previous studies [22,39], and MHC expression was upregulated in this study. Therefore, we suggest that maca intensified the expression of MHC-fast/slow under the influence of EPS even with augmented metabolism-related protein expression [22,39,63]. A recent review suggested that increased phosphorylation of AMPK activates a fiber-type transition from MHC-fast to MHC-slow expression in skeletal muscle cells [64]. In the present study, however, both MHC-fast/slow were upregulated, suggesting that not the fiber type shift but other mechanisms such as the myogenetic pathway (e.g., Pax7, Myogenin, MyoD) might be involved in these findings [38].

One limitation of this study was that the current EPS conditions did not reproduce the hypertrophic effects seen in previous studies using 2D skeletal muscle cells [42]. We think this discrepancy may be due to the long intervention time of EPS. Previous research showed that the increase in duration will lead to a significant downward trend in the effect of EPS [65]. Basically, mechanical stress is an important factor causing muscle hypertrophy in in vitro models, so, it is necessary to explore the conditions that promote muscle hypertrophy solely under EPS in the 3D-EM to mimic hypertrophic effects seen in the 2D muscle cells, such as duration and frequency of EPS, which might be important factors to mediate specific cellular signaling [24]. Nonetheless, given that the 3D-EM closely mimics the structural and contractile properties of in vivo muscles, 3D-EM is posited to serve as a valuable tool to discover and develop nutrition and exercise modality instead of conducting animal experiments; however, further refinement of this model is necessary, such as using human engineered skeletal muscle, to advance this field.

## 5. Conclusions

To sum up, maca promotes skeletal muscle hypertrophy under EPS conditions by upregulating metabolic function-related proteins in the 3D-EM. This highlights the first use of the 3D-EM as a next-generation in vitro model to study the combined impacts of exercise (i.e., EPS) and nutrition (i.e., maca), and holds promise for combining them as a preventive measure against sarcopenia.

## Data Availability

Not applicable.
